# Does substance use by family members and community affect the substance use among adolescent boys? Evidence from UDAYA study, India

**DOI:** 10.1186/s12889-021-11911-5

**Published:** 2021-10-20

**Authors:** Shobhit Srivastava, Pradeep Kumar, Rashmi, Ronak Paul, Preeti Dhillon

**Affiliations:** International Institute for Population Sciences, Mumbai, Maharashtra 400088 India

**Keywords:** Substance addiction, Tobacco and alcohol consumption, Family members, Adolescent boys, Community, Multilevel analysis, UDAYA

## Abstract

**Background:**

Substance use among adolescents is risky behavior that had emerged as a concern in both developed and developing countries. Evidence revealed that substance use is more frequent among those adolescents whose immediate family members (parents, siblings and grandparents) also indulge in such consumption; however, scarce literature is present in the Indian context. Therefore, the present study examined whether substance use among family members and in the community is associated with the substance use behavior of adolescent boys in Uttar Pradesh and Bihar.

**Method:**

We used the data for 5969 adolescent boys aged 10–19 years from the Understanding the Lives of Adolescents and Young Adults (UDAYA) survey conducted in 2016. A three-level random intercept logit model was utilized to understand the association of adolescent substance use behavior with familial and community context.

**Results:**

We found that 16% of adolescent boys were using any substance (tobacco or alcohol or drug). The substance use was significantly higher among adolescent boys who were school dropouts (40%) than those who were currently in school. The prevalence of substance use is also high among those who were working (35%). Moreover, 19, 24 and 28% of the adolescents come from families where at least one of the family members consumed tobacco, alcohol and drugs, respectively. The odds of substance use were 2.13 times [CI:1.44–3.17] higher among those adolescent boys whose family members also indulged in substance use. Moreover, the likelihood of substance use was 1.24 times [CI:1.01–1.68] higher among the adolescent boys who come from a community with high substance use. Additionally, the risk of substance use is more likely among adolescent boys belonging to the same household of the same community.

**Conclusion:**

It is evident that exposure to substance use in the family and community increases the likelihood of substance use among adolescent boys. There is a need for household- and community-level programmatic interventions to alleviate the risk of substance use among adolescents.

## Introduction

The adolescent phase involves exploring with new things along with an experience of physical, social, emotional and mental changes [1]. However, with the growing magnitude of responsibilities, experiences and competition among adolescents, the issue of risky behavior that affects the health of adolescents has received immense attention [2]. Substance use among adolescents is one such risky behavior that had emerged as a global concern [3, 4]. Substance use is defined as the use of harmful mood-altering substances like alcohol, illicit drugs, tobacco and others. Substance use turns health-abusive when taken at the repeated course, leading to deleterious health issues and impairments with the capacity to affect the body physically, mentally and socially [5]. Globally, the consumption of alcohol was alone attributable to more than 3 million deaths [6]. Of this alcohol consumption-related death burden, India contributes a larger portion of 273,000 preventable deaths [7].

According to the report “Magnitude of substance use in India”, the use of harmful substances like opioids, inhalants, sedatives, injected drugs, and alcohol had increased at an alarming rate, especially among adult Indian men [8]. In India, consumption of tobacco and alcohol has become common among adolescents, and in the case of tobacco consumption, the age of initiation was observed to be as low as 12 years [9]. Smokeless tobacco usages have also become very popular in India owing to its easy availability and inexpensive price [10]. Moreover, the use of both smokeless and smoked tobacco, alcohol and cannabis (a psychoactive drug) is widely prevalent among the adolescents residing in the Indian slums [11]. Existing studies had linked the substance usage among adolescents with age [12], education [13], poverty [14], migration and occupation exploitation [15], creating cool image among peers [16], working status [17], drug culture [18], socioeconomic correlates [19]. Moreover, substance use among the family members has also emerged to be an important predictor of substance use among adolescents in both developed and developing countries [20, 21].

Studies have revealed that substance use is more frequent among those adolescents whose immediate family members (parents, siblings and grandparents) also indulge in such consumption [22, 23]. Existing research had further shown that generational continuity [24] and perception of drinking in the family was associated with excessive drinking behavior in adolescents [25]. A systematic review of 58 research papers had indicated that smoking use among family members makes adolescents more likely to indulge in smoking behavior [20]. Further, a study had shown that substance use by older siblings increases the likelihood of substance use among younger siblings [26]. Extant research has also shown that adolescent substance use has long-term consequences in the form of – deteriorating health status, exhibiting violent behavior, proneness to accidents, loss of employment, a dropout from formal education, poor performance in education and career development, among the people of both developed [3, 27] and developing nations [28, 29].

Minimal research in India had come forward showing the effect of substance use among family members on adolescent substance use behavior. Despite having an awareness of the behavior-inducing effect of familial substance use on adolescents and the damaging consequences of such behavior, there is very limited research regarding the same in the Indian context. A single study had documented the initiation of tobacco use among those adolescents whose immediate family members (parents, grandparents or siblings) were already into it [30]. Besides the family effect, a couple of studies had also pointed towards the importance of community in adolescent substance use behavior [31, 32]. A multilevel study from United States Midwestern countries shows the influence of parents, communities, schools and peers on adolescent substance use behavior [33]. Moreover, a study from Northeast India had shown the role of community-related characteristics behind the increment of tobacco consumption among the whole population [34]. It was observed in these studies that consumption behavior varies with community culture, and thus, the role of the community behind an individual’s behavior cannot be denied.

This brings the necessity to explore how the use of tobacco, alcohol, drugs and other substances among the family members affect the adolescent’s substance use, taking into consideration the community factors. Different Indian studies have shown that the use of tobacco, alcohol and drugs was higher among adolescents [35–37]. In spite of such research, two relatively backward big Indian states (i.e., Uttar Pradesh and Bihar) lack the knowledge due to sparse public health data. A study by the National Drug Dependence Treatment Centre (NDDTC) had stated the vulnerability of adolescents in Uttar Pradesh towards the consumption of harmful substances [38]. Further, evidence reveals that boys were a highly vulnerable group among adolescents, but scarce literature shows that such vulnerability is due to familial and community contexts. This brings the point of departure for the present study. Using data from the Understanding the Lives of Adolescents and Young Adults (UDAYA) survey, this study examined whether substance use among family members and in the community is associated with the substance use behavior of adolescent boys in Uttar Pradesh and Bihar. From the methodological point of view, a three-level random intercept logit model is used to capture the effect of all individual, household and community factors on the substance use behavior of adolescent boys.

## Data, variables and methods

### Data

Secondary data analysis was performed on a cross-sectional dataset obtained from the Understanding the Lives of Adolescents and Young Adults (UDAYA) survey [39]. The survey was conducted in the two Indian states Uttar Pradesh and Bihar, in 2016 by Population Council under the guidance of the Ministry of Health and Family Welfare, Government of India. The UDAYA collected detailed information on family, media, community environment, assets acquired in adolescence, and quality of transitions to young adulthood indicators [39]. The sample size for Uttar Pradesh and Bihar was 10,350 and 10,350 adolescents aged 10–19 years, respectively. The required sample for each sub-group of adolescents was determined at 920 younger boys, 2350 older boys, 630 younger girls, 3750 older girls, and 2700 married girls in both states. The UDAYA adopted a multi-stage systematic sampling design to provide the estimates for states as a whole as well as for the urban and rural area of the states. The detailed information on the sampling procedure and survey design was published elsewhere [39]. The effective sample size for this study was 5969 adolescent boys aged 10–19 years.

### Outcome variable

The outcome variable was substance use among adolescent boys. The variable was recoded as 1 “yes” if the respondent is either consuming tobacco products or alcohol or drugs and was categorized as 0 “no” if the respondent does not consume any of the three products. The drug use was probed for the consumption of brown sugar (a heroin product), cocaine, and traditional cannabis-derivative products such as “ganja”, “charas”, and “bhang” [39].

### Predictor variables

1. Substance use among family members was coded as “no” and “yes”, i.e. if in a family anyone was consuming either tobacco products or alcohol or drugs were categorized as experiencing substance use “yes” otherwise “no,” i.e. no one is consuming either of the three substances [40].
2. Age (in years) was coded as early adolescents (10–14 years) and late adolescents (15–19 years) [40].
3. Current schooling was coded as never attended, dropout and currently attending [40].
4. Working status was coded as no and yes [40].
5. Media exposure was coded as no exposure, rare exposure and frequent exposure [40].
6. Caste was coded into Scheduled caste/Scheduled tribe (SC/ST) and non-SC/ST [41].
7. Religion was coded as Hindu and non-Hindu. The category of non-Hindu was recoded as the frequency of other religions except the Muslim religion was low [42].
8. Wealth index was coded as poorest, poorer, middle, richer and richest [43].
9. Residence was available in data as urban and rural [40].
10. States were recoded as Uttar Pradesh and Bihar [42].

### Community-level variables

Community-level variables were constructed by aggregating the individual/household-level characteristics of the respondents to the primary sampling unit (PSU) level [44, 45]. The UDAYA data provided a household wealth index (WI) based on information collected on household amenities and assets. The community economic index was divided into two categories, “high” and “low”, with “low” being for PSUs whose average household WI was less than the national average of WI and “high” is for the remaining PSUs [44, 45]. Similarly, the individual’s educational index was created based on the average years of schooling of women at the PSU level and similarly mother’s educational index was also created [44]. The community-level substance use was also created based on average media exposure at the PSU level and then dividing it into low and high as per average substance use among the individuals in a particular community [45].

### Statistical analysis

The study used univariate and bivariate analysis to show the sample distribution of the study population and the association between the outcome and predictor variables, respectively. Further, multilevel (three-level) logistic regression analysis [44] was used to assess the effects of the individual-, household-, and community-level variables on substance use among adolescent boys [45]. The random effects of household and community were estimated by using the melogit command in STATA (Version 15) [46].

The application of the multilevel modelling was justified by the hierarchal structure of the survey, where adolescents were nested within households and households were nested within primary sampling units (PSUs) [45]. First, a null model was run; that is, without keeping any explanatory variables [44]. This model represented the total variance in substance use at household and community levels [44]. In multivariate modelling, three models were fitted. In the first model, individual-level variables included, the second model included individual- and household-level variables. In the last model, community-level variables were added [45]. The fixed effects at the individual, household, and community levels, and the random effects at the household and community levels, were calculated [44, 45]. For all the estimated models, the significance of the fixed effect parameters was evaluated by using *p*-values (*p* < 0.05) [44, 45]. The mathematical description of the final model (three levels) is given below:

$$ logit\left({\pi}_{ijk}\right)=\mathit{\log}\left(\frac{\pi_{ijk}}{1-{\pi}_{ijk}}\right)={\beta}_{0 jk}+{\beta}_1{x}_{1 ijk}+{\beta}_2{x}_{2 ijk}+{\beta}_3{x}_{3 ijk}+\dots +{\beta}_n{x}_{nijk} $$

Here, *π*_*ijk*_ = *p*(*y*_*ijk*_ = 1) is the probability that adolescents (i) in the household j, from the PSU k, in the substance use. Where *y*_*ijk*_ is equal to “1” if an adolescent boy uses substance use and “0” if they did not. The study defined this probability as a function of an intercept and the exploratory variables as follows: *β*_0*jk*_ = *β*_0_ + *μ*_0*jk*_.

In this equation, β_0jk_ indicates that the paper modelled the intercept in this relationship as random at *j*^*th*^ (household) and *k*^*th*^ (PSU) levels. The variables *x*_1*ijk*_*to x*_*nijk*_ were the explanatory variables, and their coefficients were the fixed effects. The technical advantage of this methodology relies on the error term structure. Linear or logistic regression models exhibit one error term for the whole equation, whereas multilevel analysis generates one error term for each level, allowing to isolate the individual-level and group-level residual variance. The split error term in the multilevel analysis allows assessing unobserved effects at every level [47].

## Results

### Sample description

**Fig. 1:**
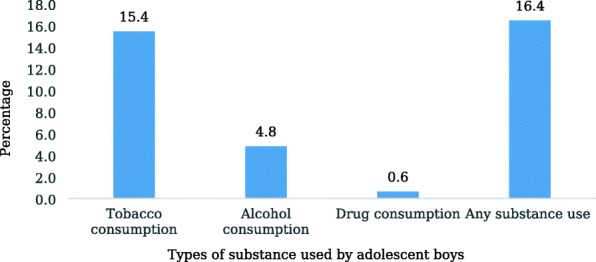
Substance use among adolescent boys aged 10–19 years

Figure 1 depicts that about 15 and 5% of adolescent boys used tobacco and consumed alcohol, respectively. Moreover, nearly 16% of adolescent boys were using any substances (tobacco or alcohol or drug).

**Fig. 2:**
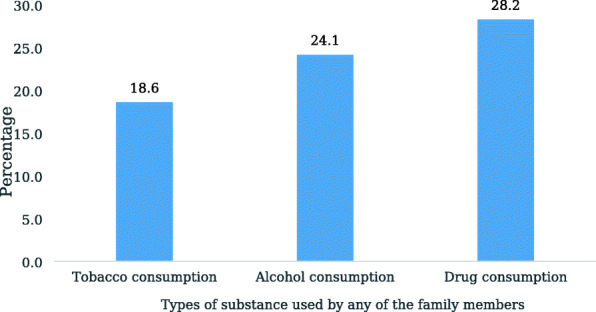
Type of substance used by the family members and its influence on substance use among adolescent boys

Figure 2 depicts that drug consumption among family members was the most important factor for adolescents to consume substance use. For instance, about 28% of adolescent boys consumed drugs, followed by alcohol (24.11%) whose family members were consuming the same.

Socio-economic and demographic profiles of adolescent boys aged 10–19 years are presented in Table 1*.* Nearly 65% of adolescent boys belonged to the late adolescent group, 18% of boys were school dropouts, and about 27% of adolescent boys were working. Nearly three-fourth of any family members of adolescent boys used any substances. Around three-fourth of adolescent boys had frequent media exposure and about a similar proportion of adolescents belonged to non-scheduled caste/scheduled tribe (SC/ST). About 31% of the community had low education, and 48% of the community belonged to the low wealth quintile.

**Table 1:** Socio-economic and demographic profile of adolescent boys aged 10–19 years

Background characteristics	Sample	Percentage
**Age (in years)**		
Early adolescents (10–14)	2084	34.9
Late adolescents (15–19)	3885	65.1
**Current Schooling**		
Never attended	190	3.2
Dropout	1092	18.3
Currently attending	4687	78.5
**Working status**		
No	4377	73.3
Yes	1592	26.7
**Substance use among family members**		
No-one	1594	26.7
Someone in the family	4375	73.3
**Media exposure**		
No exposure	335	5.6
Rarely	1078	18.1
Frequently	4555	76.3
**Caste**		
Scheduled Caste/Scheduled Tribe	1605	26.9
Non- Scheduled Caste/Scheduled Tribe	4364	73.1
**Religion**		
Hindu	5001	83.8
Non-Hindu	968	16.2
**Wealth index**		
Poorest	704	11.8
Poorer	1193	20.0
Middle	1374	23.0
Richer	1391	23.3
Richest	1308	21.9
**Community education**		
High	4125	69.1
Low	1844	30.9
**Community wealth**		
High	3118	52.2
Low	2851	47.8
**Community substance use**		
Low	2773	46.5
High	3196	53.5
**Residence**		
Urban	1030	17.3
Rural	4939	82.7
**State**		
Uttar Pradesh	4069	68.2
Bihar	1900	31.8
**Total**	5969	100.0

### Prevalence of substance use among adolescent boys

The percentage distribution of substance use among adolescent boys by background characteristics is shown in Table 2*.* The prevalence of substance use was significantly higher among late adolescents (22.9%) compared to early ones (4.5%). Adolescent boys who never attended school (38.5%) or school dropout (40.3%) had a higher prevalence of substance use, and it was lowest among those who were currently studying (10%). Working boys (35.2%) had a higher prevalence of substance use than not working ones (9.6%). The prevalence of substance use was significantly higher among adolescents with no media exposure (20.3%) compared to those who had frequent media exposure (16.4%). It was found that substance use was more prevalent among boys (18.5%) whose family members used any substances. Moreover, SC/ST (21.5%) and non-Hindu (17.2%) adolescent boys used more substances compared to their counterparts. The wealth of the family had a negative association with substance use among adolescent boys. The prevalence of substance use was significantly higher among boys (18.3%) if the community had low education. Similarly, if the community belonged to the low wealth quintile, boys used more substances (17.7%).

**Table 2:** Percentage distribution of substance use among adolescent boys by background characteristics

Background characteristics	Percentage	***p***-value
**Age (in years)**		0.001
Early adolescents (10–14)	4.5	
Late adolescents (15–19)	22.9	
**Current schooling**		0.001
Never attended	38.5	
Dropout	40.3	
Currently attending	10.0	
**Working status**		0.001
No	9.6	
Yes	35.2	
**Media exposure**		0.043
No exposure	20.3	
Rarely	15.6	
frequently	16.4	
**Substance use among family members**		0.001
No-one	10.7	
Someone in the family	18.5	
**Caste**		0.033
Scheduled Caste/Scheduled Tribe	21.5	
Non- Scheduled Caste/Scheduled Tribe	14.6	
**Religion**		0.001
Hindu	16.3	
Non-Hindu	17.2	
**Wealth index**		0.001
Poorest	21.5	
Poorer	17.6	
Middle	16.6	
Richer	16.1	
Richest	12.9	
**Community education**		0.001
High	15.6	
Low	18.3	
**Community wealth**		0.048
High	15.3	
Low	17.7	
**Community substance use**		0.002
Low	14.0	
High	18.6	
**Residence**		0.812
Urban	14.4	
Rural	16.9	
**State**		0.145
Uttar Pradesh	16.9	
Bihar	15.6	
**Total**	16.4	

### Estimates from three-level random intercept model

Estimates from multilevel logistic regression analysis for substance use among adolescent boys are presented in Table 3. Model 1 included individual-level explanatory variables such as the age of the adolescent boys, schooling, working status, and mass media exposure which all were significantly associated with substance use except media exposure. Model 2 included household-level variables in addition to the explanatory variables used in Model 1, and Model 3 added community-level predictors. Model 3 showed that late adolescents [OR: 6.65; CI: 3.06–14.45] had significantly higher odds of substance use than early ones. Boys who were currently studying [OR: 0.10; CI: 0.03–0.29] had 90% fewer odds to use any substances compared to those who never attended school. The likelihood of substance use was 4.46 times significantly more likely among working boys [OR: 4.46; CI: 2.42–8.22] compared to not working ones. Similarly, the odds of substance use among boys whose family members used any substances [OR: 2.13; CI: 1.44–3.17] was 2.13 times higher compared to their counterparts. Further, we found that substance use was 24% [OR: 1.24; CI: 1.01–1.68] more likely among those adolescent boys who come from a community with high substance use in comparison to their counterparts who come from communities with low substance use.

**Table 3:** Multilevel logistic regression analysis assessing the effect of background characteristics on the likelihood of substance use among adolescent boys

Background characteristics	Model-1	Model-2	Model-3
	OR (95% CI)	OR (95% CI)	OR (95% CI)
**Age (in years)**			
Early adolescents (10–14)	Ref.	Ref.	Ref.
Late adolescents (15–19)	7.38*(1.67,32.6)	6.81*(3.07,15.07)	6.65*(3.06,14.45)
**Current schooling**			
Never attended	Ref.	Ref.	Ref.
Dropout	0.68 (0.35,1.34)	0.74 (0.42,1.30)	0.67 (0.37,1.20)
Currently attending	0.08*(0.01,0.59)	0.11*(0.04,0.32)	0.10*(0.03,0.29)
**Working status**			
No	Ref.	Ref.	Ref.
Yes	5.09*(1.63,15.94)	4.22*(2.32,7.67)	4.46*(2.42,8.22)
**Media exposure**			
No exposure	Ref.	Ref.	Ref.
Rarely	0.88 (0.44,1.74)	0.92 (0.49,1.73)	0.89 (0.47,1.68)
frequently	1.29 (0.67,2.5)	1.40 (0.76,2.57)	1.43 (0.78,2.63)
**Substance use among family members**			
No-one		Ref.	Ref.
Someone in the family		2.22*(1.48,3.33)	2.13*(1.44,3.17)
**Caste**			
Scheduled Caste/Scheduled Tribe		Ref.	Ref.
Non- Scheduled Caste/Scheduled Tribe		0.75 (0.55,1.03)	0.72*(0.52,0.99)
**Religion**			
Hindu		Ref.	Ref.
Non-Hindu		1.09 (0.77,1.55)	1.28 (0.88,1.84)
**Wealth index**			
Poorest		Ref.	Ref.
Poorer		0.69 (0.42,1.15)	0.72 (0.44,1.19)
Middle		0.80 (0.50,1.30)	0.90 (0.56,1.45)
Richer		0.66 (0.40,1.08)	0.78 (0.47,1.28)
Richest		0.80 (0.49,1.33)	1.05 (0.62,1.78)
**Community education**			
High			Ref.
Low			0.78 (0.56,1.09)
**Community wealth**			
High			Ref.
Low			1.31 (0.90,1.92)
**Community substance use**			
Low			Ref.
High			1.24*(1.01,1.68)
**Residence**			
Urban			Ref.
Rural			1.09 (0.76,1.54)
**State**			
Uttar Pradesh			Ref.
Bihar			1.56*(1.12,2.17)

*p < 0.05; OR: Odds ratio; CI: Confidence interval, Ref: Reference categories, Model 1 included individual factors; Model 2 included household level factors along with individual; Model 3 contained all the individual, household and community level variables

A model applied without covariates (called the null model) on substance use among adolescent boys (Table 4) showed a significant amount of variation in the prevalence of substance use across families and communities. Based on the intra-class correlation coefficient (ICC) values, about 11% and 6% of the total variance in the prevalence of substance use were attributable to differences across families and communities, respectively. After including individual (Model 1), household (Model 2) and community-level variables (Model 3) in the null model, the ICC value decreased to 5% at the community level and increased to 56% at the household level.

**Table 4:** Variance estimates across families and communities, and the intra-class correlation coefficient for the multilevel models for substance use among adolescent boys

Random Effect Parameters	Null	Model 1	Model 2	Model 3
Community (PSU) random variance (SE)	0.20 (0.06)	0.57 (0.39)	0.40 (0.20)	0.35 (0.17)
Household random variance (SE)	0.18 (0.39)	5.09 (6.32)	3.83 (2.83)	3.78 (2.76)
Community (PSU) ICC (%)	5.7	6.0	5.4	4.7
Household ICC (%)	10.7	63.1	56.3	55.6

## Discussion

The present study uses the UDAYA survey to examine the association of substance use behavior by adolescent boys and their family members in Uttar Pradesh and Bihar. It was evident that the adolescent boys were higher likely to indulge in substance use if at least one of their family members also indulged in substance use. Further, substance use was common among those adolescent boys who have experienced high substance use in the community they come from. We also found a high degree of correlation in the propensity for substance use within the same household and the same community. These findings point towards the role played by constant environmental exposure to substance use within a household and in the surrounding community, which inculcates the habit of substance use among inquisitive adolescents.

Analyses revealed that adolescent boys of Uttar Pradesh and Bihar were more inclined towards tobacco consumption behavior. Thus, the behavior not only questions the different tobacco policies introduced in India but shows the ill effect of the easy availability and inexpensive nature of such products among Indian adolescents [10, 34]. Moreover, drug consumption among adolescent boys was only 0.6%, but its consumption among their family members seems to be the substance that is more influential for them. Further, the association of adolescent boys substance use with their family was consistent with a study from India which had also revealed that in familial settings, if parents, grandparents, or elder siblings frequently ask the adolescent boys of the family to fetch tobacco-based substances or alcohol, then the boy indulges in early usage of such substances [13]. Moreover, adolescents may perceive this behavior as a tradition after watching their elders. However, it is often found in Indian tradition that younger individuals are not expected to use such substances in front of their elders; this restriction can also create the curiosity to try different substances secretly among adolescents.

Indeed, family members’ consumption behavior was predictive of adolescent’s substance use habits. Multilevel analyses revealed the importance of the environment in which an adolescent resides, which was consistent with the findings of previous research [33]. The role of family and community cannot be denied and was found to be influential as the risk of substance use is more likely among adolescent boys belonging to the same household of the same community. It should be noted that households and communities can have both pros and cons on adolescent’s behavior. Although the present study found that individuals sharing the same characteristics in certain households and communities paving the way for substance use among adolescent boys. But introducing community-driven programs can also help in curbing such behavior among adolescent boys. Moreover, sensitization of family members can further help in the reduction of substance use behavior of adolescent boys. Evidence of higher adolescent substance use is observed among the working population, which is consistent with an Indian study [48]. The present study shows boys in the late adolescence period are more prone to substance use. Such association brought forward the role of providing knowledge about harmful effects of substance use among adolescent right from the early phase, so that they may not get indulge in such behavior later or while their peer exposure during work. A higher level of formal education among adolescents had an influential role in keeping them away from different substance use, indicating the role of education in an adolescent’s life. The adolescent boys who were attending school were less likely to use any substance, and this finding was consistent with an existing Indian study [13]. Another study from high school Indian adolescents had a consistent finding with this study which shows that media can increase substance use behavior by providing a frequent source of provoking advertisements [49]. However, the results were contradicted with a study that shows both pro and anti-effect of media on humans health [50]. These individual factors have also detrimental effects when seen in the family and community context.

Using data from the two Indian states (i.e., Uttar Pradesh and Bihar), this study had tried to fill the knowledge gaps from these states about the determinants of substance use among adolescent boys. Present study utilizes the data available on familial and community contexts in Uttar Pradesh and Bihar which are often limited in other surveys. Moreover, the survey gathered information from adolescent boys regarding their usage of different substances like tobacco-, drugs- and alcohol-based products. Unlike in other Indian surveys, this helped us not to stick to any particular consumption behavior. Existing literature showing the effect of the family-level risk factors is mostly based on the developed countries [31–33]. Only a few existing studies from India had talked about the effect of the family members’ consumption behavior on adolescents of the family. However, our study has examined the effect of both family-level and community-level factors and provided clear evidence of substance use risk among adolescent boys belonging to the same household of the same community. The results of this study are backed by few studies of developed and developing countries and expand our knowledge towards adolescent substance use.

However, it is also important to consider a few limitations of this study. This study did not consider important school- and peer-related characteristics of adolescents like academic performance, indulged in a bad peer group, faces violence, or bullying. One study had found evidence of academic performance and peer influence with adolescent substance use [51]. Also, a longitudinal or panel data study is required to notice the behavior of adolescents at later ages. Nested associations should also be noticed in different ages and regions. Moreover, confined to data of Uttar Pradesh and Bihar, these results cannot be generalized to that of the whole nation. However, it does provide interesting insights into the substance use pattern of adolescent boys from the two high-prevalence Indian states.

## Conclusion

Despite some limitations, this study highlights the relevance of family risk factors on adolescent boy’s substance use behavior. Moreover, the study shows that among 16% of adolescent boys indulged in substance use behavior, tobacco consumption (15.4%) had increased at an alarming rate. Community substance use among family members had also emerged as an important indicator of the growing adolescent boy’s substance use behavior. Our findings re-emphasize the need to bring the children for schooling education and target those adolescents who were unable to continue their education and are forced to work for livelihood in early and late adolescence period. Further, the evidence, of substance use is more common among adolescent boys belonging to the same household of the same community, provided additional support to this study and turned the attention towards targeting these factors. The study had highlighted the need for interventions, which target the adolescent boys right from their household.

## Data Availability

Data was collected as part of Population Council’s UDAYA study which is publicly available on the site of Harvard Dataverse at https://dataverse.harvard.edu/dataset.xhtml?persistentId=doi:10.7910/DVN/RRXQNT

## Abbreviations

OR: Odds Ratio
CI: Confidence Interval
UDAYA: Understanding the Lives of Adolescents and Young Adults
SC: Scheduled Caste
ST: Scheduled Tribe
